# Predicting conserved protein motifs with Sub-HMMs

**DOI:** 10.1186/1471-2105-11-205

**Published:** 2010-04-26

**Authors:** Kevin Horan, Christian R Shelton, Thomas Girke

**Affiliations:** 1 Department of Computer Science and Engineering, University of California Riverside, Riverside, California, USA; 2 Department of Botany and Plant Sciences, University of California Riverside, Riverside, California, USA

## Abstract

**Background:**

Profile HMMs (hidden Markov models) provide effective methods for modeling the conserved regions of protein families. A limitation of the resulting domain models is the difficulty to pinpoint their much shorter functional sub-features, such as catalytically relevant sequence motifs in enzymes or ligand binding signatures of receptor proteins.

**Results:**

To identify these conserved motifs efficiently, we propose a method for extracting the most information-rich regions in protein families from their profile HMMs. The method was used here to predict a comprehensive set of sub-HMMs from the Pfam domain database. Cross-validations with the PROSITE and CSA databases confirmed the efficiency of the method in predicting most of the known functionally relevant motifs and residues. At the same time, 46,768 novel conserved regions could be predicted. The data set also allowed us to link at least 461 Pfam domains of known and unknown function by their common sub-HMMs. Finally, the sub-HMM method showed very promising results as an alternative search method for identifying proteins that share only short sequence similarities.

**Conclusions:**

Sub-HMMs extend the application spectrum of profile HMMs to motif discovery. Their most interesting utility is the identification of the functionally relevant residues in proteins of known and unknown function. Additionally, sub-HMMs can be used for highly localized sequence similarity searches that focus on shorter conserved features rather than entire domains or global similarities. The motif data generated by this study is a valuable knowledge resource for characterizing protein functions in the future.

## Background

The identification of functionally relevant features in protein sequences is an important task for gaining insight into their molecular and biological activities. Commonly used feature classifications systems focus on protein regions of different lengths ranging from single residues in active site representations and relatively short sequence motifs to much longer protein domains. The identification of these functional modules is often of immediate importance for guiding molecular and evolutionary studies of genes and genomes, such as experimental or computational discoveries of drug targets, catalytic residues and ligand binding sites [1-6]. Due to the greater evolutionary constraints, the functionally important regions in proteins tend to be more conserved among related sequences than their less relevant regions. As a result of this basic similarity-function principle, one can predict the functional features in proteins relatively reliably by identifying their conserved regions [7,8]. The same information is often useful to predict differences of the catalytic and substrate specificities within subgroups of protein families by identifying their specificity determining residues [9,10].

Profile hidden Markov models (profile HMMs) provide the basis of very efficient approaches for modeling longer conserved regions in protein families, which are referred to as protein domains [11-14]. These domain models usually co-align well with longer functional and structural units of proteins, such as protein folds [15,16]. The genome regions coding for protein domains, rather than entire genes, are often considered the functional base units of protein evolution. Because domain models are relatively complex by covering longer conserved sequence areas, the identification of essential sub-features within protein domains can greatly facilitate their functional characterization. Well known examples are the conserved protein motifs from the PROSITE database [17,18]. These much shorter patterns frequently map to residues within protein domains that are directly involved in the core functions of many proteins, such as the coordination of the catalytic centers of enzymes. The most specific and functionally insightful information about known or predicted active sites is provided by protein structure-based resources, such as the Catalytic Site Atlas (CSA), CASTp, ActSitePred, ConSurf and PDBSite [7,19-23]. The utility spectrum of these structure-based resources is typically restricted to proteins that share sequence similarity with proteins of known 3D structure. This requirement of structure information makes these methods less suited for functional site predictions of many membrane proteins or other difficult to crystallize protein classes. Thus, it is important to develop additional tools that can be used for the prediction of functionally relevant features of all protein classes. Conservation analyses are widely used alternatives for this purpose [8,24-26]. Typically, these methods aim to identify conserved residues in multiple sequence alignments of related proteins. Based on the above principle, these conserved sites tend to be functionally more important than more variable ones. More recently developed approaches incorporate additional information with conservation data, such as secondary structure predictions, solvent accessibility data and other parameters [27,28]. In addition, Mistry *et al*. [22] have developed a set of strict rules that allows the transfer of experimentally validated active site information to other sequences within the same enzyme family. A disadvantage of most conserved residue approaches is the difficulty of using their data sets without major modifications for search applications in order to identify novel proteins containing these features. The more information rich motif and domain models are usually more effective in this regard. This is also facilitated by the availability of many efficient motif or domain search algorithms in this area.

Much of the information available in conserved sequence databases is the direct result of mining the available protein space with existing feature prediction tools. This includes very established databases on protein motif or domain information, such as PROSITE, InterPro and Pfam [2,4,18]. However, the annotation and curation process of the conserved features provided by these databases is still a very time consuming and largely manual curation processes by many experts in the field. Therefore, the development of additional functional feature prediction methods, that can facilitate the automation of various steps in this laborious annotation process, will be of great importance for the field.

Here we propose an automated method for identifying conserved protein motifs by creating sub-HMMs from custom or existing profile HMM data sets, such as Pfam. The method builds on existing profile HMM domain models and expands their utility spectrum to motif discovery. The approach has many applications for studying protein functions. First, it is useful for predicting the most highly conserved and functionally relevant sequence motifs in protein families. Second, it provides an effective alternative for profile-based similarity searches to detect sequences with short similarities in any order. Finally, it can be used for the characterization of domains of unknown function by associating them with sub-HMMs from functionally characterized domains.

The most closely related method for modeling protein families by a fragment-based approach was proposed by Plotz and Fink [29]. Their goal was to minimize the number of parameters used by the model in order to improve its performance on small training sets. To achieve this, the authors started with a signal-like protein sequence representation [30] and trained a new model on this data set. Their model consisted of Sub-Protein Units (SPUs) that were concatenated in an order learned from the data set. Each SPU of this method is an HMM by itself. In contrast to this, our method uses pre-calculated profile HMMs to discover functionally relevant motifs in protein domains. In addition, our method has the ability to allow any combination of sub-HMMs to occur in any order. Another related method is Meta-MEME [31]. This method also minimizes the number of model parameters. It accomplishes this by concatenating short PSSMs (Position Specific Scoring Matrix) instead of HMMs, which are generated by its sister tool MEME [32]. This approach is similar to the BLOCKMAKER program [33], which also models conserved regions with un-gapped PSSMs. Our method differs from these approaches significantly by retaining full HMMs of the most highly conserved sub-regions within protein domain families. This allows us to model more complex consensus regions containing gaps. The method developed by Sun and Buhler [34] attempts to speed up searching with profile HMMs by extracting un-gapped subsections (blocks) of HMMs and then modifying the match distributions in each position to make each block as sensitive as possible. These blocks are then used as pre-filters to eliminate sequences which would not match the whole HMM well.

Our proposed *protein sub-HMM *method starts with a profile HMM that has been trained on the multiple sequence alignment of a protein family. We then extract the most conserved sub-HMMs from the original HMM. A robust scoring method is used to predict the presence of the sub-HMMs in any protein sequence of interest. The HMMs required for this approach can be easily generated from unaligned protein sequences of interest by aligning them with a multiple sequence alignment program and then generating an HMM for them with tools like HMMER [14,35,36] or SAM [37]. Alternatively, one can use existing protein family HMMs from databases like Pfam [38]. The latter approach is taken in this paper for benchmarking the proposed protein sub-HMM method.

## Results and Discussion

**Figure 1:**
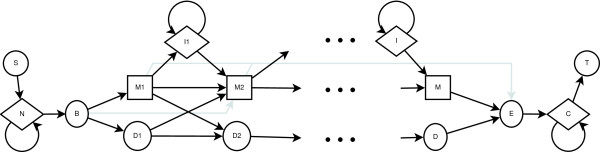
**PLAN 7 HMM**. The profile HMM of a multiple sequence alignment is illustrated, using the PLAN 7 model in HMMER2. State transitions are illustrated by arrows, match states by squares, insert states by diamonds and delete states by circles.

A profile hidden Markov model of a sequence family is a statistical model over sequences whose structure consists of a number of states and transitions between states. For each state *z *there is a distribution, *P*(*x*|*z*) over a set of observations, *x *∈ *X*. In our case, *X *is the set of amino acids. A transition matrix *T*(*z*_1_|*z*_2_) defines the probability of transitioning from state *z*_2 _to state *z*_1_. We can view this transition matrix as a graph in which a link exists from *z*_2 _to *z*_1 _if *T*(*z*_1_|*z*_2_) > 0. Figure 1 shows the structure used for aligning protein sequences [35]. For each nominal position *i *there are three possible states: a match state *M*_*i*_, an insert state *I*_*i*_, and a delete state *D*_*i*_. *P *(*x*|*M*_*i*_) is a distribution over amino acids occurring at position *i*. *P*(*x*|*I*_*i*_) is a background distribution, which is the probability of each amino acid occurring given no other information. This state is used to model noise sections in the input sequence. The delete state does not have a real observation distribution; it requires that nothing be observed (an ϵ observation). This is used to model sections of the input sequence which have been lost.

The parameters of an HMM can be learned using the Expectation Maximization (EM) [39] algorithm given a set of observed protein sequences (but not the hidden state sequence), producing a model tuned to this set of protein sequences. Once the model has been trained, we can take another protein sequence, *S*, and ask what is the most likely sequence of HMM states to generate *S*, and what is the probability of that combination of states and observations. This is done with the Viterbi algorithm [40]. To rank the results, it is common to calculate the log-odds:(1)

In this equation, *P*_back_(*S*) is the probability of *S*, assuming each amino acid has been drawn independently from the background distribution, while *P*_HMM_(*S, Z*) is the probability that the HMM would generate the state sequence *Z *and the observed sequence *S*. A positive score means that *S *is more likely to be derived from the HMM than randomly generated from the background distribution. A more detailed description of profile HMMs can be found in [41].

### Extraction of Sub-HMMs

**Figure 2:**
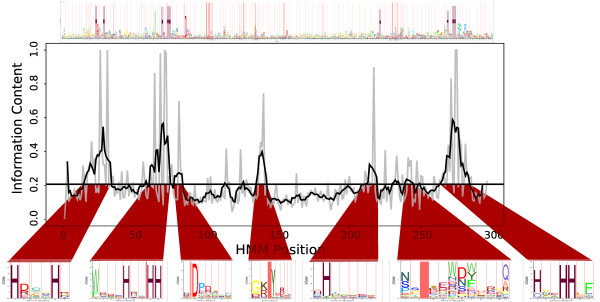
**Sub-HMM Extraction Process**. An example of the sub-HMM excision process is given for the fatty acid desaturase domain (PF00487). The light gray line is the KL-divergence of each position in the original HMM. The darker line is the result of smoothing with *s *= 8. The horizontal line is the threshold, set to the average KL-divergence. Each section of the curve with more than *l *= 8 positions above the threshold produces a sub-HMM. The details about the different parameters are given in the Method section.

Our sub-HMM method is built on top of the well-established profile HMM framework described above. The algorithm consists of a simple but effective two step procedure for extracting the most highly conserved regions from profile HMMs (compare Figure 2). First, the Kullback-Leibler divergence is calculated for all columns of a profile HMM [42]. Second, after a series of normalization and smoothing steps (see Methods section), the most information rich HMM regions are excised from the original profile HMMs. The resulting sub-HMMs have the same structure as the original profile HMMs, but they are usually much shorter. Typically, the method will extract several non-overlapping sub-HMMs from a single domain model, especially when its most conserved regions are highly localized and discontinuous. A more detailed outline of the algorithm for extracting sub-HMMs and using them for scoring their presence in protein sequences is described in the Methods section. In the following outline we first describe our sub-HMM experiments and provide several performance comparisons to related tools. Subsequently, we use our tool to find sequences that share short sequence features encoded in our sub-HMMs.

### Properties of Sub-HMMs

Sub-HMMs were extracted from Pfam domain families using HMMER2 and HMMER3 models [43]. Pfam 22.0 was used for all experiments, whereas Pfam 24.0 was mainly used in the performance comparisons with HMMER3. This is because Pfam has adopted HMMER3 models only very recently, and at this point many of its families have not been as rigorously tested and curated by experts in the field as in the earlier HMMER2-based releases.

**Figure 3:**
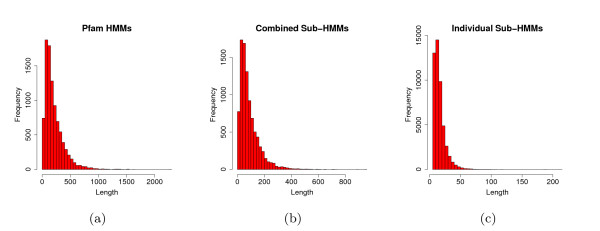
**Length Distribution of Pfam HMMs and Sub-HMMs**. (a) The length distribution of Pfam HMMs is depicted in the form of a histogram. The Pfam HMMs consist on average of 210 positions, while it is only 90 positions for the combined set of sub-HMMs per Pfam HMM. (b) The length per domain model is computed by summing the lengths of the sub-HMM extracted from that model. (c) The length distribution of individual sub-HMMs is shown. In all cases the sub-HMMs were created with a minimum length setting of 8 and a smoothing factor of 8.

Using our new sub-HMM method, we extracted 48,535 sub-HMMs (Table 1) from the Pfam 22.0 database (Pfam-A, Pfam_ls). This database consisted of 9,318 domain profile HMMs with 2,990,695 unique protein sequences associated with at least one domain. Due to the presence of multiple domains in many sequences, the data set contained a total of 4,070,949 family memberships. The length distributions of the original Pfam HMMs and our sub-HMMs for all families are shown in Figure 3. As expected the sub-HMMs are much shorter than the original Pfam HMMs, with an average length of 17 residues compared to 210 residues, respectively. This has several advantages for the goals of this study. First, the sub-HMMs have a length distribution similar to the size of many known functional motifs, which is essential for predicting features with related properties [17,18]. Second, their shorter length reduces the computation time for scoring a protein. Finally, it reduces the number of parameters, which should improve the accuracy of the detector.

**Table 1:** Data sets.

Name	Size	Description
Pfam proteins	2,990,695	Proteins in Pfam database
Pfam HMMs	9,318	Domains in Pfam database
DKFs	7,435	Pfam domains of known function
DUFs	1,883	Pfam domains of unknown function
Sub-HMMs	48,535	Sub-HMMs excised from Pfam domains
Sub-DKFs	39,217	Sub-HMMs excised from DKFs
Sub-DUFs	9,318	Sub-HMMs excised from DUFs

The table provides the sizes of the different data sets used and generated by this study using Pfam 22.0.

Subsequently, we performed several benchmark tests to determine the performance of the new sub-HMM method in identifying functionally relevant sequence features and searching for sequences sharing them. For this, we determined the presence of each Pfam HMM and our sub-HMMs in all protein sequences from the Pfam database by applying the scoring system described in the Materials section. We found that the processing time of our method is comparable to HMMER2. The slightly better time performance of our method by a factor 1.4 is most likely due to the lower complexity of its sub-HMM models. The sub-HMM method showed comparable time improvements when using it with the HMMER3 software.

### Cross-Validation with PROSITE and CSA

Next, we determined how well the sub-HMM method performed in identifying known motifs that are likely to be of functional relevance. This was addressed by comparing the extracted sub-HMMs from the Pfam 22.0 database with the hand curated conserved protein motifs from the PROSITE database. If the sub-HMMs are enriched in functionally relevant candidates, then one would expect a high degree of overlap with the motifs from the PROSITE database. This should be the case because the PROSITE motifs are derived from a comparable protein knowledge space as the sub-HMMs generated by this study. The overlaps were determined by comparing the matching positions of the two fragment data sets in their corresponding protein family sequences. For counting overlaps, we used relatively conservative filtering criteria: the two fragment models had to have 50% of their matching protein sequences in common and the overlaps had to occur in least 95% of the common protein members. In addition, we consider a sub-HMM to match only if it has a score of 0 or higher. Furthermore, we compute the probability of this event happening by chance and require that it be less than 0.01.

**Figure 4:**
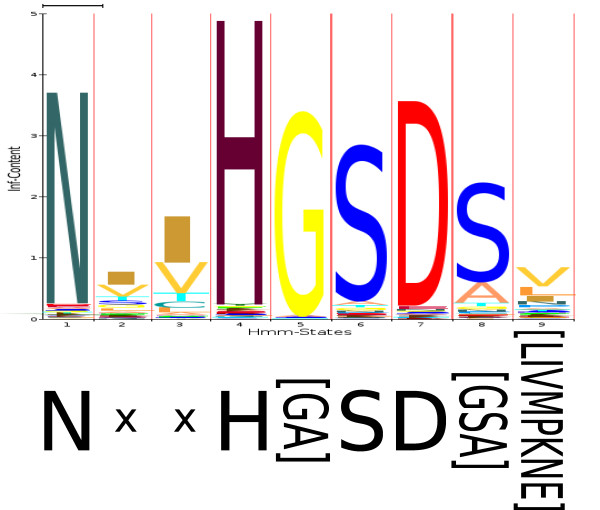
**Example of a sub-HMM matching a PROSITE pattern**. An example of a sub-HMM matching with a PROSITE pattern. In this case, the sub-HMM (PF00334.10-3, top) extracted from domain PF00334.10 matched against PS00469 (bottom) with a p-value of less than 10^-315^.

According to these comparisons, 1,055 of the 48,535 sub-HMMs overlapped with 937 of the 1,303 (72%) PROSITE motifs by at least 10% of the length of the shortest fragment. The probability of finding ≥937 matches just by chance was estimated to be < 1.6 * 10^-6 ^(see Method section for details). Of these 1,303 PROSITE motifs, 958 were associated by Pfam with one or more of its protein families. The number of matching families for varying percent overlaps is shown in Table 2. An example of a matching pair is shown in Figure 4. The full result set is available in Additional file 1:prosite-comp.tar.

**Table 2:** PROSITE Comparisons.

Overlap	Sub-HMMs	PROSITE	TP	TPR
**10%**	**1,054**	**937**	**562**	**0.58**
25%	1,023	932	558	0.58
50%	965	912	549	0.57
75%	849	827	495	0.51
90%	720	716	423	0.44
100%	620	624	366	0.38

The numbers of sub-HMMs are listed that overlapped with PROSITE motifs. The first column provides the relative overlap among the two feature types. The second and third columns contain the number of overlapping sub-HMMs and PROSITE motifs, respectively. The details of the filter settings used in these comparisons is given in the Result and Discussion section. The column TP contains the number of true positives that we identified out of the 958 PROSITE families annotated by Pfam 22.0. The last column TPR gives the corresponding true positive rate.

A similar test was performed for the catalytic residue annotations from the Catalytic Site Atlas (CSA) [19]. This is a database of active site residues from enzymes represented in the Protein Data Bank (PDB). Due to their functional importance, most of these residues are highly conserved within protein families. In our tests, we considered only those sites which are supported by the literature and also mapped to protein domain regions in the Pfam data set. This left us with 4147 sites mapping to 642 proteins. Subsequently, we counted how many sub-HMMs overlapped with these sites and found that 847 sub-HMMs overlapped with CSA residues. These corresponded to 2903 active sites from 546 proteins. Thus, our sub-HMM data set contained 70% of these active sites. The probability of observing ≥2903 overlaps among the two data sets just by chance is < 1.5 * 10^-18^. The complete result set of this analysis is available in Additional file 2:csa-comp.

The considerable agreement of our method with the PROSITE and CSA data sets indicates that the sub-HMM method is efficient in identifying many of the known functionally important residues in protein families. Therefore, it is reasonable to assume that the novel conserved regions, identified by this study, are a useful resource for characterizing the functional hotspots in protein sequences of known or unknown function in the future.

### Search Performance Comparisons

**Figure 5:**
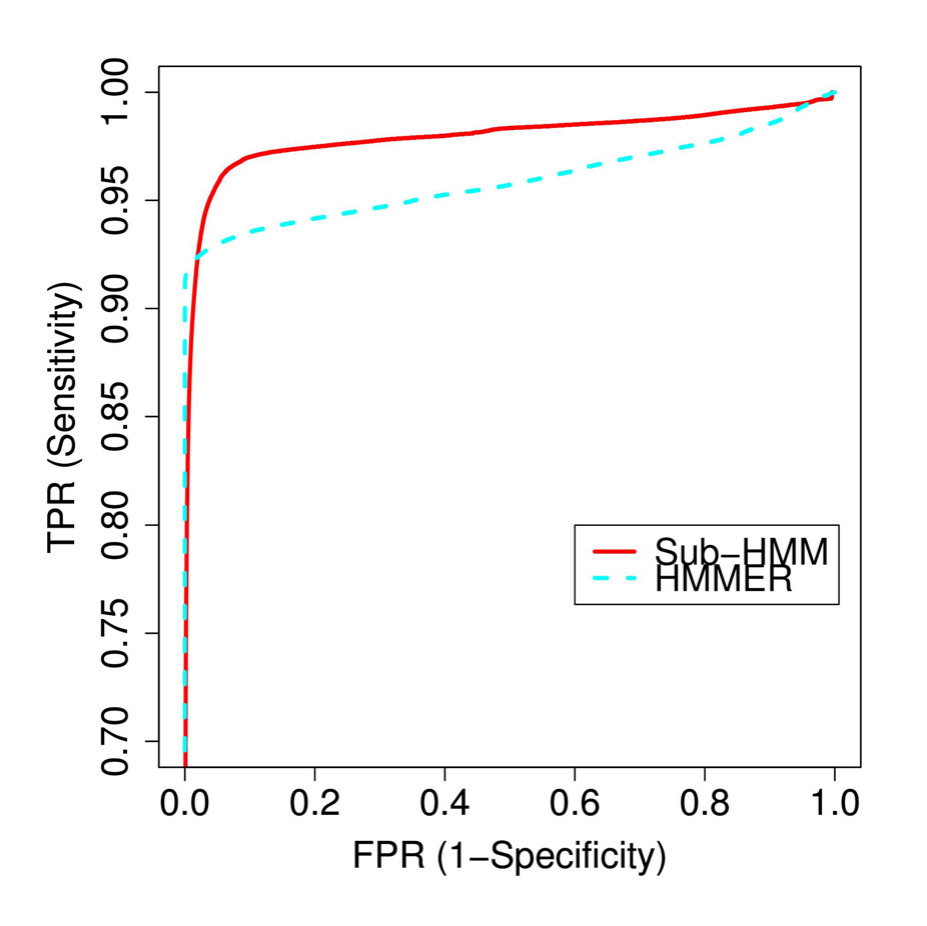
**Sensitivity versus Specificity Performance**. The true positive rates versus false positive rates are plotted in the form of ROC (Receiver Operating Characteristic) curves to compare the performance of the HMMER2 and sub-HMM methods. The full Pfam 22.0 data set was considered in this test.

To compare the sensitivity and selectivity performance of the sub-HMM method with the widely used HMMER2 software, we tested how well each method could recover the members of each domain family from all proteins in the entire Pfam 22.0 database. We used the scores computed for each protein to generate an ROC (Receiver Operating Characteristic) curve for each method (Figure 5). This allowed us to compare the methods without choosing a fixed threshold, which is usually hard to define *a priori*. In this preliminary test, we used the original Pfam HMMs for the HMMER2 method, and the sub-HMMs extracted by our method from the same Pfam HMMs. As a test sample, all proteins in Pfam were used. This experimental design gives a slight advantage to both methods, because the Pfam HMMs are trained on a representative subset of proteins that overlaps with the total protein set in each family. Despite this limitation, the difference in performance is still meaningful due to the identical starting conditions for both methods. Figure 5 shows the resulting ROC curves for assembling all 9,318 families. The results show that the HMMER2 method has a higher sensitivity at false positive rates less than 0.02, but the sub-HMM method performs slightly better at higher false positive rates. Due to the much shorter profiles used by our method, it is expected to have a higher false positive rate when it is benchmarked against a test data set that is based on the family assignments of complete domain models.

We also performed more rigorous comparisons of our method against HMMER2, HMMER3, SAM and PSI-BLAST [44]. Additionally, we tested our sub-HMM method with HMMER3 profile HMMs. In this case the sub-HMMs where excised from HMMER3 models and the HMMER3 search tool was used to map and score the individual sub-HMMs to the sequences. We then combined the scores as described in the Methods section. In the following text of this section, the sub-HMM experiments performed with HMMER2 and HMMER3 are referred to sub-HMM-HMMER2 and sub-HMM-HMMER3, respectively. In all tests we trained the models ourselves by randomly selecting 20% of the members from each protein family, but the training data were not included in the test data sets. HMMER2, HMMER3 and SAM use a multiple sequence alignment for the model building step. Since it was not our goal to test the alignment quality, we used the curated domain alignments provided by Pfam as input to all methods. Although SAM can create its own alignments, we forced it to use the alignments we provided to make this method more comparable to HMMER2 and HMMER3. For PSI-BLAST, we first created multiple sequence alignments for all the training data sets using CLUSTALW. Subsequently, we built PSSMs to search the test data set with PSI-BLAST. For all methods, we compared how well they could recover the remaining 80% in each protein family from the combined set of all test sequences. Due to computational resource constraints, it was not possible to test these methods on all Pfam families. Instead we created two smaller subsets of families, one composed of smaller families and one composed of larger families. The small family set contained 933 families randomly selected from Pfam 22.0 with of 10 to 100 members, while the large set contained 1002 families with more than 100 members. In addition, we tested the different methods on the HMMER3-based Pfam 24.0 data set. To maximize the comparability of the results, we selected only families that were available in both Pfam releases and fell into the same size categories. For the small set, we found 899 families in Pfam 24.0 but only 491 of them had less than 100 members. For the large set, 988 families were also available in Pfam 24.0 and all of them contained more than 100 members.

**Figure 6:**
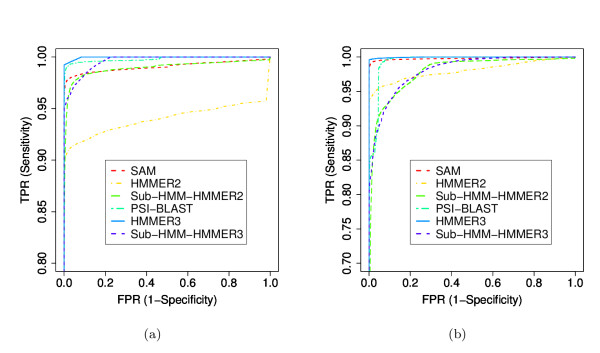
**Sensitivity versus Specificity Performance on Small and Large Families from Pfam 22.0**. The performances of HMMER2, HMMER3, SAM, PSI-BLAST, sub-HMM-HMMER2 and sub-HMM-HMMER3 on the Pfam 22.0 data set are compared in the form of ROC curves (compare Figure 5). The first test (a) considers smaller families with 10 to 100 members, whereas the second one (b) considers large families with more than 100 members.

**Figure 7:**
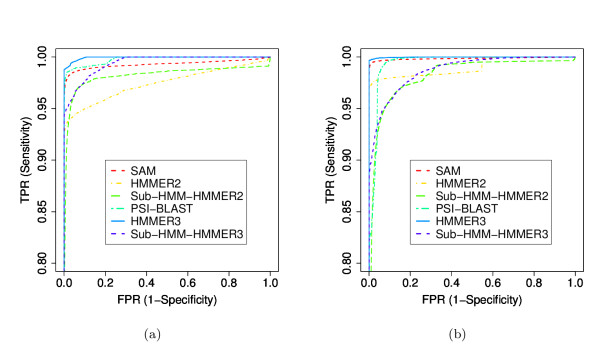
**Sensitivity versus Specificity Performance on Small and Large Families from Pfam 24.0**. Performance comparisons with Pfam 24.0. The first test (a) considers families used for Figure 6 that were also present in Pfam 24.0 for the small (a) and the large sets (b).

The ROC plots for all comparisons are shown in Figures 6 and 7. For the experiments with Pfam 22.0, the results indicate that the sub-HMM-HMMER2, sub-HMM-HMMER3 and PSI-BLAST methods perform better on the small family set than on the large one, while HMMER2, HMMER3 and SAM show an opposite performance trend. When comparing the six methods, both sub-HMM methods perform at least as well as HMMER2, whereas SAM, HMMER3 and PSI-BLAST show the best performance in assembling the families from both family size categories. Direct comparisons of the Pfam 22.0 and Pfam 24.0 results indicate that HMMER3, PSI-BLAST, SAM, sub-HMM-HMMER2 and sub-HMM-HMMER3 perform very similarly on the small family set, while HMMER2 improves slightly. These trends are almost identical for the large family set, except that sub-HMM-HMMER3 performs better on this data set.

Since our method is designed to find short sequence similarities, it is expected to have a lower selectivity (higher false positive rate) than the other methods when reassembling family relationships that are based on longer domain similarities. In fact, such a performance characteristics on known family data sets is required for discovering novel conserved fragments in sequences that do not necessarily belong to the same domain family. The latter is the main utility feature of the sub-HMM method.

### Discovery of Conserved Fragments in Protein Families with Sub-HMMs

To evaluate the utility spectrum of sub-HMMs for conserved feature discovery, we determined for each sub-HMM excised from Pfam 22.0 its matching profile against different domain families in the same Pfam release. To define a match, we required a sub-HMM to match at least 50% of the sequences in each Pfam family with a log-odds score of 0 or higher. Table 3 shows how many sub-HMMs from Pfam domains of unknown function (DUFs) matched Pfam families of known function (DKFs) and vice versa. A sub-DUF is defined as a sub-HMM that was extracted from a DUF, whereas a sub-DKF was extracted from a DKF. Interestingly, the sub-DKFs shows considerable overlaps with the PROSITE data set, whereas the sub-DUFs do not overlap with PROSITE at all (last two rows in Table 3). The latter is expected because PROSITE focuses on motifs from functionally characterized proteins. This also indicates that our sub-DUF data sets contains many novel conserved and potentially functional motifs that are not represented in PROSITE.

**Table 3:** Matches Among DUFs and DKFs.

Match Type	Sub-HMMs	Pfam HMMs	OL PROSITE/Sub-HMM
sub-DKF → DKF	28,794	6,571	689
sub-DKF → DUF	21,615	1,751	502
sub-DUF → DKF	6,798	5,487	0
sub-DUF → DUF	5,070	1,516	0

The table lists the numbers of sub-DKFs and sub-DUFs which matched in addition to their source families other DKF and DUF families. A sub-HMM is considered to have matched a Pfam 22.0 family if it scores greater than 0 on more than 50% of its members. The last column contains the counts of sub-HMMs that also overlapped with PROSITE motifs.

**Figure 8:**
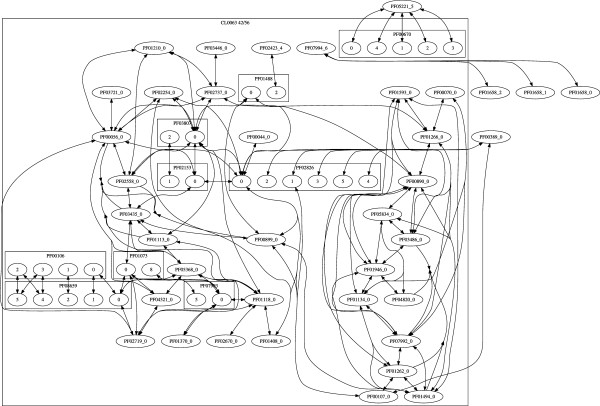
**Pfam Clan Comparison**. The graph shows an example of a Pfam clan and the corresponding sub-HMM network. The sub-HMM method clusters Pfam domains by conserved fragments. In the given example, the results from both methods agree very well. The Pfam clan membership is indicated by the large box labeled CL0063. The oval nodes with a PF* label represent domain families for which only one sub-HMM was created. The rectangular boxes labeled with a PF* number represent that domain family and nodes inside are sub-HMMs created from that family.

**Figure 9:**
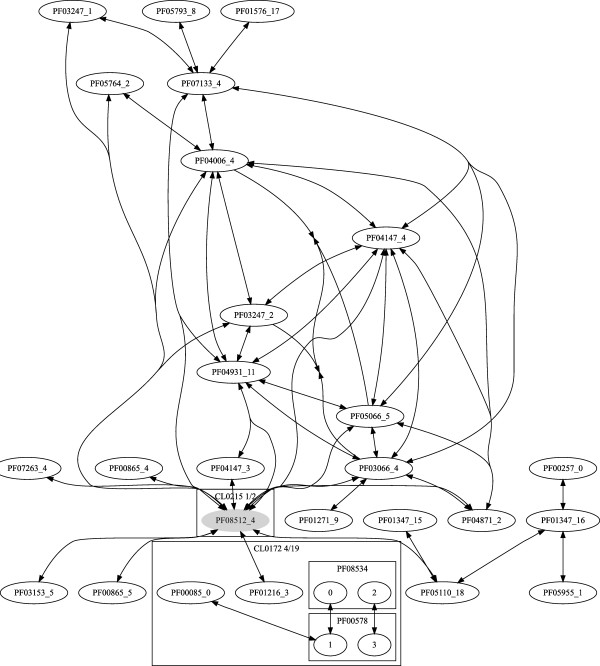
**Domain Cluster Predicted by Sub-HMM Method**. An Example is given for a novel Pfam domain cluster that could be predicted by the Sub-HMM method. The dark nodes indicates a domain of unknown function (DUFs). The other symbols in the graph are explained in the legend of Figure 8.

A similar approach was used for constructing networks of Pfam 22.0 families by their common sub-HMM matches. The obtained clusters in this network showed many similarities to the clusters from the Pfam clan database, but also significant differences [3]. The Variation of Information (VI) coefficient [45] for the two network sets was 0.275. This score has a range from 0 to log(9318) = 9.1, with lower scores indicating more similar clusterings. Two small sub-graphs of the sub-HMM based domain network are shown in Figures 8 and 9. The box in Figure 8 encloses those families which are part of a clan according to the Pfam database. In this case the sub-HMM-based grouping of families agrees almost perfectly with the corresponding Pfam clan assignment. In contrast to this, Figure 9 gives an example of a new cluster of domains predicted by our method. Such differences in the results of the two methods are expected, because the Pfam clans are assembled with a profile HMM to profile HMM alignment method [46] that is fundamentally different from our sub-HMM method.

The large number of sub-HMMs matching different Pfam domains indicates the usefulness of our sub-HMM approach for discovering short sequence features that are conserved among different protein domains. Due to their high conservation, an important functional role for many of these features can be expected. Many of the sub-DKFs will be useful for assigning potential functions to DUFs. A much more comprehensive study on applying our sub-HMM approach to biologically relevant questions will be published in an experimental journal.

## Conclusions

We have developed a simple but effective method for identifying the most highly conserved residues in protein sequences in a fully automated manner. Its design strategy is highly practical and versatile by making efficient use of a well-established bioinformatic infrastructure, such as existing domain databases and profile HMM search tools. In addition, the conserved patterns, identified by this study, are useful for characterizing proteins of unknown function by associating them with those of known function by their common sub-HMMs. Furthermore, the sub-HMM search method appears to be a very effective tool for finding sequences that share only very short sequence similarities with a sensitivity performance similar to HMMER2. The possibility to ignore the order of different sub-HMM matches in sequences is another advantage, which will allow the identification of more complex similarity arrangements among otherwise unrelated sequences.

## Methods

### Extracting sub-HMMs from Profile HMMs

To extract the desired sub-HMMs from a single profile HMM, *H*, with length *H*_*l*_, we first compute the Kullback-Leibler divergence (or relative entropy) [42] of each position in the original HMM:(2)

Here *M*_*i *_is the observation distribution of the match state at position *i*, and *B *is the background distribution. We normalize *h *by dividing by the maximum value, so that each position has a value between 0 and 1, and then smooth the values:(3)

**Figure 10:**
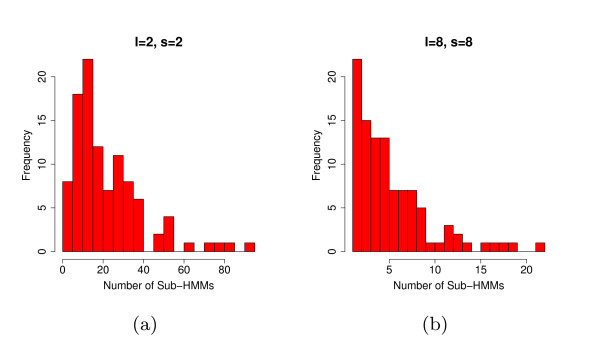
**Number of generated Sub-HMMs**. (a) The number of sub-HMMs generated from 100 families with a minimum length (*l*) of 2 and a smoothing factor (*s*) of 2. (b) The number of sub-HMMs generated from 100 families with parameters *l *= 8 and *s *= 8. Lower values of *s *and *l *produce more and shorter sub-HMMs, while higher values produce fewer and longer sub-HMMs. This result is typical for all families.

The smoothness of the curve is determined by parameter *s*, with higher values producing a smoother curve. Let  be the set of ranges of *h*^*s *^that are always above threshold *t *and at least *l *positions long. For each member of *L *we extract positions *m *through *n *+ *s *of *H*. The endpoint *n *is extended by *s *because  includes information about positions *n *through *n *+ *s *of *H *that we want to maintain. We always set *t *to be the average over *h*. Finally, we examine each extracted sub-HMM and trim off positions at the beginning and end for which . Figure 2 shows an example of this process. The extracted sub-HMMs are themselves full HMMs and have the structure shown in Figure 1. Finding the best values of *s *and *l *is difficult. However, through experimentation we found that setting both values to 8 works well for sub-HMM-HMMER2, while *s *= 15 and *l *= 8 works best for sub-HMM-HMMER3. When *s *is increased, more positions with low relative entropy will be incorporated into sub-HMMs resulting in more specific models. Such models will tend to only match very similar protein fragments. Small *l *values will increase the number of sub-HMMs, whereas the opposite trend is observed for larger *l *values. An example of these differences is shown in Figure 10.

Once the consecutive regions of match states are identified from the original profile HMM, we convert each of them into a sub-HMM. Each sub-HMM has the same structure, transition probabilities, and observation distributions as the corresponding segments in the source HMM. As the original HMMs, the sub-HMMs begin and end with looped insertion states. Typically, a sub-HMM obtained from this process is identical to a profile HMM trained on the corresponding region of a multiple alignment that was used for generating the original profile HMM.

### Scoring of Sub-HMM Matches

Sub-HMMs can be matched and scored against protein sequences either as single models or as sets of models. When scoring a set of sub-HMMs against a protein sequence *S*, such as all sub-HMMs extracted from a Pfam HMM, we used a method based on a complete generative model. We hypothesize the entire protein sequence can be generated according to the following sampling semantics: First, choose the length of the sequence. Then, for each sub-HMM *y*, sample the starting location from a uniform distribution, and then sample a sequence from *y *and place it at the chosen starting point. After this is done for all the sub-HMMs, fill in the gaps with samples from the background distribution. This assumes that each of the sub-HMMs generates a portion of the protein sequence, while their order is not important. In addition, we ignore possible overlaps among sub-HMMs. We use the Viterbi algorithm to find, for each sub-HMM, the most likely hidden state sequence and position in *S*, using a local-local alignment. Let *M *be the length of *S *and *Y *the set of sub-HMMs. Then the resulting score is:(4)

Here score_*y*_(*S*) is the score from Equation 1 for HMM *y*. The term |*Y*| log *M *arises from the uniform distribution over positions at which any sub-HMM might begin. We implemented our method in Java and used code from HMMEditor [47] to run the Viterbi algorithm. This score can be computed in time linear in *M *and the combined lengths of the sub-HMMs.

In the ROC performance tests, we scored sequences using sub-HMMs grouped by the Pfam families they were excised from. For all other tests, we scored individual sub-HMMs by using score_*y*_(*S*) as the final score.

### PROSITE and CSA Comparisons

The overlaps of sub-HMMs and PROSITE motifs were computed by matching them against the domain sequences in each Pfam family. The PROSITE matches were determined with ps_scan [48]. To minimize the compute time of these overlap comparisons, we considered only those Pfam and PROSITE sets (families) which had at least 50% of their sequences in common. Among these, at least 95% of the matches had to overlap by variable lengths specified in Table 3. The overlaps with the CSA data set were computed similarly. Due to the short length of the active sites, their positions had to be completely contained in the sub-HMM matches. The probability of a sub-HMM matching with a PROSITE motif by chance was computed as follows. We let *q*_*ij *_be the probability that a sub-HMM match of length *F*_*j *_overlaps a PROSITE match of length *P*_*j *_on a protein of length *S*_*j *_from a Pfam family *i *by a fraction of at least *x*:(5)

Then we compute the probability, *D*_*i*_, that a certain number of overlaps occurs between a sub-HMM and a PROSITE motif within a given Pfam family *i*. Let *F *be the set of sequences in a Pfam family and *P *the set of sequences in a PROSITE family. We define ℛ as the set of all subsets of *F *∩ *P *which contain at least 95% of the intersection:(6)

where *n *= |*F *∩ *P*|. Let *p*_*ij *_= {*q*_*ij*_|*j *∈ *F *∩ *P*}, then:(7)

Since the enumeration of every set in ℛ is time intensive, we approximate it with an upper bound. Let j* = argmax_*j*_*p*_*ij*_, then we have:(8)

In equation (9), we replace the sum from the previous equation with the sum over the possible sizes of *R*. For each size, the binomial term gives the number of sets of size *k*, and the last term gives the probability of a set of size *k*. However, this bound is often too loose in practice. This is because for large values of *p*_ij*_, the last term in equation (7) makes that term very small, whereas the corresponding term in our bound would still be large. Therefore, we adopt a method of removing extreme outliers to obtain a tighter bound.

In the end we have:(10)

where *n' *is the number of elements remaining in the intersection after the outliers have been removed. More details about this method are provided in Additional file 3:prosite_scoring.pdf.

We use the Hoeffding bound [49] to upper-bound the likelihood of finding a certain number of PROSITE or CSA overlaps with our sub-HMM data set by chance (that is, if the sub-HMM data set had instead been chosen at random). The Hoeffding bound states that if the random chance of any single test matching is *p*, then the probability of *m *or more matches in *M *tests is less than  where .

For the PROSITE comparisons, matches are only considered if the prior probability is less than 0.01, therefore, *p *= 0.01. We found *m *= 1,055 overlaps out of a total set of *M *= 48, 535 sub-HMMs. This yields a p-value (by the Hoeffding bound) of less than 1.6 * 10^-6 ^for the probability of our sub-HMMs matching PROSITE models at this level by chance.

For the CSA comparison, each site is only a single amino acid. We restrict the comparisons to only those sequences containing annotated CSA sites. There are *M *= 95, 076 amino acids matching our sub-HMMs, of which *m *= 2, 903 are annotated by CSA. There are a total of 261, 857 amino acids, of which 4, 147 are annotated by CSA. Therefore, , and we obtain (again with the Hoeffding bound), a p-value of less than 1.5 * 10^-18 ^for the probability of our sub-HMMs overlapping these CSA-annotated amino acids by chance.

### ROC Comparisons

For the PSI-BLAST tests, the training sets were aligned with CLUSTALW [50] and then a PSSM was generated using blastpgp with just one round of searching. The test data was then scored by blastpgp using the trained PSSM as a starting point and running for up to 6 rounds. For each sequence, we recorded the maximum log-odds score from all the rounds. For the SAM tests, we extracted the aligned training data from the Pfam database and used them to train the models, forcing SAM to use the given alignments rather than create its own. These models were then used to classify the test data. In the case of HMMER2 and HMMER3, we trained models with hmmbuild and hmmcalibrate (HMMER2 only) using the same alignments as for the SAM tests. In all cases, HMMER2 tests were performed with HMMER2 models and HMMER3 tests with HMMER3 models. We then used these models to classify the test data with hmmsearch. If multiple domains were found in one sequence, the result from the best scoring one was used.

For the sub-HMM method, we used the aligned training data to build HMMER2 and HMMER3 models, and then extracted sub-HMMs from them. We then used our hmmsearch implementation to score each sequence according to our model. For all tests, the training sets consisted of a random selection of 20% of the sequences from each Pfam family, while the test database contained the union of the remaining sequences. The ROC curves where computed with the ROCR library [51] using the concatenation of all the scores for each method. Log-odds scores were used for all methods to obtain comparable results. In the case of SAM, we used reverse log-odds scores [52].

### Availability of Software and Data Sets

The sub-HMM software developed by this project is available for free download from our web page: http://subhmm.ucr.edu. The site also contains download options of the complete set of extracted sub-HMMs and data for the Pfam network analysis, as well as a searchable web interface.

## Authors' contributions

KH performed the experimental work and contributed to writing the manuscript. CS devised the sub-HMM model and edited the manuscript. TG proposed the research problem, designed the test experiments and wrote the manuscript. All authors have read and approved the manuscript.

## Supplementary Material

Additional file 1 **PROSITE Comparisons**. Tar archive containing a file for each overlap threshold given in table 2. Column descriptions are given at the beginning of each file. Click here for file

Additional file 2 **CSA Comparisons**. Plain text file containing data for the comparison between CSA and sub-HMMs. Column descriptions are given at the beginning of the file. Click here for file

Additional file 3 **P-Value Calculations**. A detailed description of computing the p-value for the comparison to PROSITE. Click here for file
