# Supplementary material for: Predicting conserved protein motifs with Sub-HMMs
Source: BMC Bioinformatics. 2010 Apr 26;11:205. doi: 10.1186/1471-2105-11-205 (PMC2879284; doi:10.1186/1471-2105-11-205)
Supplement: Additional file 3 — P-Value Calculations. A detailed description of computing the p-value for the comparison to PROSITE. [file 1471-2105-11-205-S3.PDF]

Calculation of p-values for sub-HMM/PROSITE overlaps

Let q_{ij} be the probability that a query fragment of length F_j overlaps a PROSITE fragment of length P_j on a protein of length S_j in PFAM family i by a fraction of at least x:

$$q_{ij} \leq \min\left(1, \frac{P_j + F_j - 2x\min(P_j, F_j) + 1}{S_j - F_j + 1}\right) \tag{1}$$

Then we want to compute the probability, D_i, that a certain number of overlaps occurs between a PFAM family i and a PROSITE family. In particular, given that at least 50% of the members of either family lie in the intersection, we want the probability that 95% of the sequences in the intersection have an overlapping fragment.

Let F be a PFAM family and P be a PROSITE family. We define $\mathcal{R}$ as the set of all subsets of $F \cap P$ which contain at least 95% of the intersection:

$$\mathcal{R} = \{R | R \subset F \cap P \wedge |R| \geq 0.95n\} \tag{2}$$

where $n = |F \cap P|$. Let $p_{ij} = \{q_{ij} | j \in F \cap P\}$, then

$$D_i = \sum_{R \in \mathcal{R}} \left(\prod_{j \in R} p_{ij} \prod_{j \in (F \cap P) \setminus R} 1 - p_{ij} \right) \tag{3}$$

Since this would require enumerating every set in $\mathcal{R}$, this would take too long to calculate, so we approximate it with an upper bound. Let $j^* = \text{argmax}_j p_{ij}$ and $R^* = \text{argmin}_R(|R|)\ \forall R \in \mathcal{R}$. Then we have

$$D_i \leq \sum_{R \in \mathcal{R}} \prod_{j \in R} p_{ij} \tag{4}$$

$$\leq \sum_{R \in \mathcal{R}} p_{ij^*}^{|R|} \tag{5}$$

$$\leq |\mathcal{R}| p_{ij^*}^{|R^*|} \tag{6}$$

$$= \left(\sum_{k = \lceil 0.95n \rceil}^{n} \binom{n}{k} \right) p_{ij^*}^{\lceil 0.95n \rceil} \tag{7}$$

This bound is often too loose in practice however. This is because for large values of p_{ij^*}, the last term in equation 3 makes that term very small, whereas the corresponding term in our bound would still be large. Therefore, we adopt a method of removing these large outliers to get a tighter bound.

$$D_i = \sum_{R \in \mathcal{R}} \left(\prod_{j \in R} p_{ij} \prod_{j \in (F \cap P) \setminus R} 1 - p_{ij} \right) \tag{8}$$

$$= \sum_{R \in \mathcal{R}} \left(\prod_{\substack{j \in R \\ j \leq n'}} p_{i(j)} \prod_{\substack{j \in R \\ j > n'}} p_{i(j)} \prod_{j \in (F \cap P) \setminus R} 1 - p_{ij} \right) \forall n' \in [1, n] \tag{9}$$

$$= \min_{n' \in [1,n]} \sum_{R \in \mathcal{R}} \left(\prod_{\substack{j \in R \\ j \leq n'}} p_{i(j)} \prod_{\substack{j \in R \\ j > n'}} p_{i(j)} \prod_{j \in (F \cap P) \setminus R} 1 - p_{ij} \right) \tag{10}$$

To simplify the notation, we re-write this in terms of the following sets:

$$U = F \cap P \tag{11}$$
$$U_- = \{x | x \in U \wedge p_{ix} \leq p_{in'}\} \tag{12}$$
$$U_+ = U \backslash U_- \tag{13}$$
$$\mathcal{R}_- = \{S | S \subset U_- \wedge |S| \geq n' - 0.05n\} \tag{14}$$
$$\mathcal{R}_+ = 2^{U_+} \tag{15}$$

These essentially divide U and $\mathcal{R}$ into their corresponding sets for elements less than $p_{in'}$ and elements greater than $p_{in'}$. Now we can rewrite Equation (10) as:

$$\leq \min_{n' \in [1,n]} \sum_{S^- \in \mathcal{R}_-} \sum_{S^+ \in \mathcal{R}_+} \left(\prod_{j \in S^-} p_{ij} \prod_{j \in S^+} p_{ij} \prod_{j \in U_- \backslash S^-} 1 - p_{ij} \prod_{j \in U_+ \backslash S^+} 1 - p_{ij} \right) \tag{16}$$
$$= \min_{n' \in [1,n]} \sum_{S^- \in \mathcal{R}_-} \left(\prod_{j \in S^-} p_{ij} \prod_{j \in U_- \backslash S^-} 1 - p_{ij} \right) \sum_{S^+ \in \mathcal{R}_+} \left(\prod_{j \in S^+} p_{ij} \prod_{j \in U_+ \backslash S^+} 1 - p_{ij} \right) \tag{17}$$
$$= \min_{n' \in [1,n]} \sum_{S^- \in \mathcal{R}_-} \left(\prod_{j \in S^-} p_{ij} \prod_{j \in U_- \backslash S^-} 1 - p_{ij} \right) \tag{18}$$

The last step follows because the last sum in Equation (17) is over every subset of U_+, so it sums to 1. We can then bound this expression as follows:

$$\min_{n' \in [1,n]} \sum_{S^- \in \mathcal{R}_-} \left(\prod_{j \in S^-} p_{ij} \prod_{j \in U_- \backslash S^-} 1 - p_{ij} \right) \leq \min_{n' \in [1,n]} \sum_{S^- \in \mathcal{R}_-} \prod_{j \in S^-} p_{ij} \tag{19}$$
$$\leq \min_{n' \in [1,n]} \sum_{S^- \in \mathcal{R}_-} (p_{in'})^{|S^-|} \tag{20}$$
$$\leq \min_{n' \in [1,n]} \sum_{k = n' - \lfloor 0.05n \rfloor}^{n'} \binom{n'}{k} p_{in'}^k \tag{21}$$

In equation (21), we replace the sum in the previous equation with a sum over the possible sizes of R. For each size, the binomial term gives the number of sets of size k, and the last term gives the probability of a set of size k.
